# Slipjoint Attachment to Handpiece for Moistening Grindstone

**Published:** 1907-06-15

**Authors:** David Stern

**Affiliations:** Cincinnati, Ohio


### Slipjoint Attachment to Handpiece for Moistening Grindstone.

BY DAVID STERN, B. S., D. D. S., CINCINNATI, OHIO.

Where the dentist uses a fountain spittoon, this appliance can be made a part of the saliva ejector and water syringe attachments, which usually accompany the spittoon. A very small stop-cock to control the flow of the water, is connected with these attachments, and an eighth-inch rubber tubing connects this stop-cock with a metallic tubing, which is slipped onto the handpiece whenever its use is indicated. This tubing is drawn out to a fine point, and by regulating the stop-cock, it allows the water to drip, drop by drop upon the grindstone, as it is applied for grinding tooth structure for any purpose.

